# Preparation and Characterization of Magnesium Alloy Containing Al_2_Y Particles

**DOI:** 10.3390/ma11091748

**Published:** 2018-09-17

**Authors:** Zhongtao Jiang, Jun Feng, Qiaowang Chen, Shan Jiang, Jiahong Dai, Bin Jiang, Fusheng Pan

**Affiliations:** 1 Research Institute for New Materials Technology, Chongqing University of Arts and Sciences, Chongqing 402160, China; jiangtao6364@163.com (Z.J.); fjfeng1994@163.com (J.F.); chenqiaowang1999@163.com (Q.C.); 2 College of Mechanical and Electrical Engineering, Yangtze Normal University, Chongqing 408100, China; daijiahong@cqu.edu.cn; 3 College of Materials Science and Engineering, National Engineering Research Center for Magnesium Alloys, Chongqing University, Chongqing 400044, China; fspan@cqu.edu.cn; 4 Chongqing Academy of Science and Technology, Chongqing 401123, China

**Keywords:** Mg-Y alloys, microstructure, Al_2_Y, heterogenous nucleation

## Abstract

A magnesium alloy containing Al_2_Y particles was successfully fabricated by changing the content of Al in the Mg-6Y alloy melt. Its microstructure and mechanical properties were subsequently characterized. The results show that two types of Al_2_Y particles were discovered in the Mg-6Y-*x*Al (*x* = 0.5–5) alloys, which are namely the polygonal particles in the pre-precipitated phase and the discontinuous network of particles in the eutectic phase. With an increase in Al content, the amount of pre-precipitated Al_2_Y increases and the eutectic decreases gradually. When the Al content is 5 wt %, Al_2_Y particles are almost all in the pre-precipitated phase in the Mg-6Y alloy. After hot extrusion, the YA65 alloy could be regarded as the Mg master alloy that contains Al_2_Y particles with heterogeneous nucleation capability or Al_2_Y particle-reinforced magnesium matrix composites. The tensile strength of the as-extruded magnesium alloy is significantly improved at ambient temperatures.

## 1. Introduction

Magnesium (Mg) alloys have attracted worldwide interest for applications involving automotive, aerospace industries, 3C products, etc., due to their multiple benefits, such as low density (1.74 g cm^−3^), high specific strength and environmental friendliness. However, the poor ductility, low creep resistance and insufficient strength at room and elevated temperatures limit their development and applications to a great extent [1,2,3,4].

Alloying is a commonly used method to improve the strength of alloys. Furthermore, grain refinement is also considered to be one of the most effective methods for enhancing both strength and ductility [5,6,7,8]. Among the currently available grain refinement methods, inoculating the heterogeneous nucleation particles is a very convenient and efficient way for foundry alloys. One of the most successful examples of this approach is the application of Al–5 wt % Ti–1 wt % B (all composition percentages are in weight throughout this current paper) master alloys in Al alloys due to TiB_2_ and Al_3_Ti particles acting as heterogeneous nucleation sites [9,10]. In fact, the Al-5Ti-1B master alloy is the Al alloy containing TiB_2_ and Al_3_Ti particles. However, for the grain refinement of Mg alloys, although the Mg-Zr master alloy containing Zr particles is a very powerful grain refiner for Al-free alloys [11,12], there is still no satisfactory commercial refiner available for Mg-Al-based alloys. Therefore, cheaper and efficient grain refiners for Mg alloys are still being studied. More recently, the published studies have suggested that the addition of alloying elements to an alloy matrix can form a secondary phase in situ that acts as a heterogeneous nucleation agent to refine grains. For example, the Mg_24_Y_5_ phase in the Mg-9Li-0.3Y alloy [13], the AgZn_3_ phase in the Zn-4.5Ag alloy [14] and the Al_3_Zr phase in the Al-0.3Zr alloy [15,16], especially the Al_2_RE series phases in the Mg-RE-Al (RE=Y, Gd, Sm, Ce or Nd) alloys [17,18,19,20,21], have been widely studied. It is also believed that these secondary phases can improve the strength of the Mg alloys due to their fine grain size and secondary phase strengthening.

Al_2_Y particle, which was formed in situ in the Mg-10Y cast alloy by adding 0.6–1.2 wt % of pure Al, is a great promising grain refiner [17,22,23]. However, the method of in-situ formation limits the composition of the Mg matrix. Furthermore, it is not clear whether Al_2_Y particles have universality in the grain refinement of other magnesium alloys. More importantly, the Mg master alloy with Al_2_Y particles that act the same as Al-5Ti-1B [24] in Al alloys has not been obtained.

The present work aims to prepare the Mg alloy that only contains granulous Al_2_Y. By studying the effects of Al content on the type, size, distribution of Al_2_Y particles in the as-cast Mg-6Y alloy and subsequent extrusion, the Mg alloy rod containing Al_2_Y particles with the capability of heterogeneous nucleation was prepared successfully. It is potentially hoped that the results may be useful for the further works that should try to develop a new Mg alloy grain refiner.

## 2. Materials and Methods

### 2.1. Preparation of the Alloys

The as-cast Mg-6Y-*x*Al (*x* = 0.5, 1, 2, 3, 4 and 5) alloys investigated in this work were prepared from pure Mg (>99.9%) (Shanxi wenxi yinguang Magnesium Industry Group Co., Taiyuan, China), pure Al (>99.9%) (South west Alwminium Industry Group Co., Chongqing, China) and Mg-20 wt % Y master alloy. All the alloys were melted in a mild steel crucible in an electrical resistance furnace under the protection of a mixed gas of CO_2_ (99 vol %) and SF_6_ (1 vol %). To ensure stabilization and obtain a homogeneous composition, the alloy constituent mixtures were held at 750 °C for 30 min. After this, the melts were poured into the cylindrical steel mold 85 mm in diameter 200 mm in height that was preheated to 200 °C. The actual chemical compositions of the experimental alloys were inspected by XRF 800CCDE X-ray fluorescence spectrometer (Shimadzu Co., Shanghai, China). The corresponding results are listed in Table 1. The cylindrical samples with a diameter of 80 mm and height of 60 mm were machined from the as-cast YA65 alloy, before the as-cast YA65 alloy ingot was homogenized at 400 °C for 10 h. The extrusion processes were conducted with a ram speed of 5 mm/s, extrusion temperature of 400 °C and extrusion ratio of 25:1 to obtain the as-extruded rod. 

### 2.2. Microstructure Characterization

The microstructural analyses of the alloys were examined by a scanning electron microscope (SEM, TESCAN VEGA 3 LMH, TESCAN Co., Brno, Czech) equipped with an energy-dispersive X-ray spectrometer (EDS; Oxford Instrument Technology Co., Ltd., Oxford, UK) system and optical microscope (OM, ZEISS Axiovert200 MAT, Carl-Zeiss Co., Yarra, Germany). The identification of any intermetallic secondary phases was achieved by using the X-ray diffractometer (XRD, D/Max 2500PC, Dandong Fangyuan InstrumentCo., Ltd., Dandong, China). In order to identify the main characteristics of the phase formation during solidification time, a thermal analysis method was used to detect their cooling curves during solidification. Before cooling, a thermocouple was placed at the center of the mild steel crucible with its tip set at 10 mm from the bottom of the crucible. The cooling curves were recorded by a datalogger and computer. Metallographic samples were ground, polished and subsequently etched with a solution containing 42 mL of ethanol, 7 mL of acetic (17.5 mol/L) and 3 g of picric acid. The grain sizes were examined by an optical microscope under polarized light and were measured by a linear intercept method.

The tensile testing of the as-extruded YA65 alloy was carried out using a universal material machine (NEW SANSI CMT-5105, XinSanSi (Shanghai) Enterprise Development Co., Ltd., Shanghai, China) with a strain rate of 3 mm min^−1^ at room temperature. The gage dimensions of tensile specimens were 5 mm in diameter 200 mm in length. The tensile direction was parallel to the extrusion direction. Tests were repeated three times for each sample.

## 3. Results and Discussion

### 3.1. Microstructure of the As-Cast Alloys

**Figure 1:**
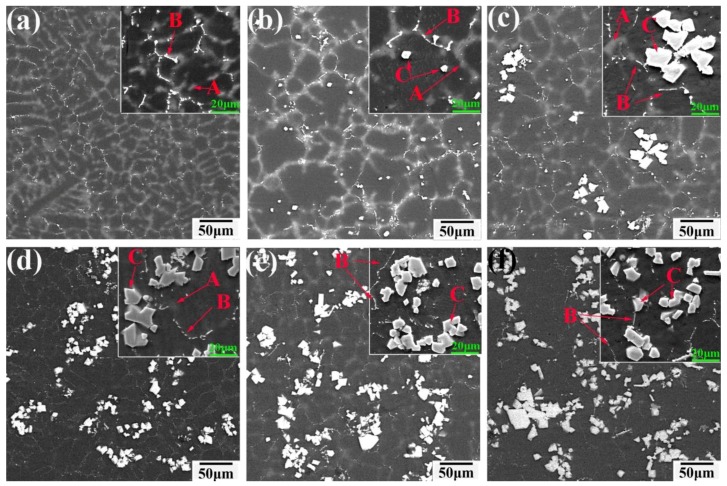
BSE-SEM images of the as-cast Mg-6Y alloys with the different addition Al contents: (**a**) 0.5%; (**b**) 1%; (**c**) 2%; (**d**) 3%; (**e**) 4%; (**f**) 5%.

The SEM images of the as-cast Mg-6Y-*x*Al (*x* = 0.5–5) alloys in Figure 1 show that as the Al contents increased, the morphology, quantity and type of the second phases in alloys changed significantly. When the proportion of the added Al is 0.5%, the discontinuous network phase along the dendrite boundary exists in the Mg-6Y alloy (Figure 1a). The polygonal particle phase begins to appear in the Mg-6Y-1Al alloy (Figure 1b). With further increases in Al content, the polygonal particles phase increases, while the discontinuous network phase reduces gradually. When the proportion of added Al is 5%, most of the Mg-6Y-5Al alloy is in the polygonal particle phase, as shown in Figure 1f. In addition, the size and distribution of the polygonal particle phase changes as the Al content increases. At first, the particle size is uniform, averaging about 5.3 μm, and it is mainly distributed inside the grains of the Mg-6Y-1Al alloy. As the Al levels rise, the distribution of the polygonal particle phase tends to an aggregation and the size increases (Figure 1b–f). In the YA65 alloy (Figure 1f), the size of the particle is in the range of about 5–18 μm.

**Figure 2:**
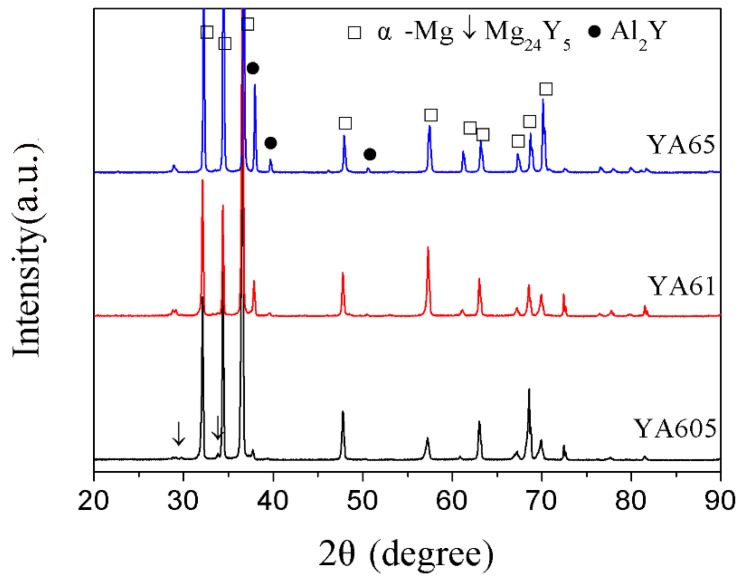
XRD patterns of the as-cast alloys.

**Figure 3:**
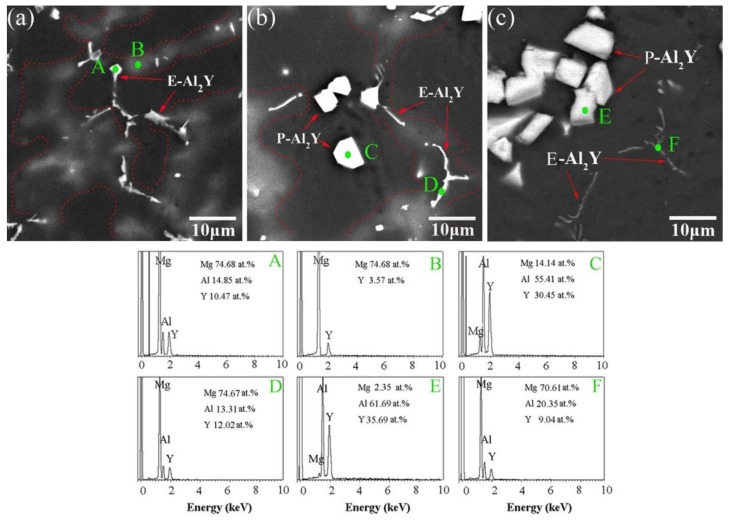
BSE-SEM image and EDS analysis of point A, B, C, D, E and F in SEM image of the as-cast: (**a**) YA605 alloy; (**b**) YA61 alloy; (**c**) YA65 alloy.

In order to determine the phase composition of the alloys, the as-cast Mg-6Y alloys with different Al additions were examined by XRD and the patterns are shown in Figure 2. It can be seen that the as-cast Mg-6Y-0.5Al alloy mainly consists of α-Mg, Al_2_Y and a small Mg_24_Y_5_ phase. When the Al content increases to 1%, the Mg_24_Y_5_ phase disappears and the only second phase is Al_2_Y in the Mg-6Y-1Al alloy. This result is consistent with that reported in literature [23]. Furthermore, when the content of Al increased to 5%, the second phase of the alloy is still Al_2_Y but the intensity of the pattern peak of the Al_2_Y phase is enhanced. As shown in Figure 1, it can be seen that when the Al content is 1–5%, the type of the second phase is the same in the Mg-6Y alloys. Therefore, only the YA61 and YA65 alloys were examined. To further discriminate the second phase in the alloys, the three magnification images of the typical microstructure and the corresponding EDS results are shown in Figure 3. The results reveal that the discontinuous second phase along the dendritic boundary (labelled as A in Figure 3a) is composed of Mg, Al and Y elements in the as-cast YA605 alloy. Combining with the XRD pattern (Figure 2), the discontinuous second phase is Al_2_Y. However, the EDS results show that the atom ratio of the Al/Y is about 1.4 (14.85/10.47).

It is important to note that there is also a grey phase along the dendritic boundary (the area marked with red dashed lines), as shown in Figure 3a,b. Furthermore, the discontinuous Al_2_Y phase is surrounded by the grey phase. The EDS result show that the grey phase is the result of the enrichment of Y elements with a content of 3.57 at % (labelled as B in Figure 3a). In addition, the EDS test was conducted with a spot size of 5 μm. Thus, the value of Y element (labelled A) is higher as the test value of point A is actually the value of the area with A as the center and 5 μm as the radius. The actual atomic ratio of the Al/Y at A point is: 14.85/(10.47 − 3.57) ≈ 2. The above results clearly show that the bright white phase of the labelled A is indeed Al_2_Y. Similarly, the bright white phase (labelled as D in Figure 3b) in the Y61 alloy also is Al_2_Y. However, the EDS of the similar phase (labelled as F in Figure 3b) in the YA65 alloy was different from that of the other two alloys. The EDS results show that the Al/Y atomic ratio is about 2. Furthermore, there is no grey phase in the YA65 alloy. The EDS combined with XRD results indicate that the polygonal particle phase (labelled as C in Figure 3b) is found in Al_2_Y. Thus, it can be seen that there are two types of Al_2_Y phases in the alloys. According to the report of Qiu et al. [13,17], the polygonal Al_2_Y particles may be also called pre-precipitated Al_2_Y (P-Al_2_Y for short). The discontinuous Al_2_Y along the dendritic boundary is speculated to be eutectic Al_2_Y (E-Al_2_Y for short).

Furthermore, the Y content in the Mg matrix, which is essentially the solution yttrium, has also been detected by the EDS (point scanning mode, each alloy has at least five points). The results are shown in the Table 2. It can be seen that as the content of Al increases, the content of the solution yttrium decreases in the alloy. Therefore, Y element exists in the alloy in the form of a compound. In general, the tendency of elements to form stable compounds was positively correlated with the electronegativity difference between elements. The electronegativity values of Y, Mg and Al were 1.22, 1.31 and 1.61, respectively [25], from which it could be deduced that Y was prone to react with Al to form Al-Y compound. Thus, the Al_2_Y phase increased gradually as Al increased, while the solution yttrium decreases in Mg matrix. In addition, it can be seen from Figure 1, with increase in Al content, the number of the P-Al_2_Y gradually increases and the E-Al_2_Y decreases gradually. Until the Al content is 5%, the as-cast YA65 alloy consists of α-Mg, P-Al_2_Y and small amounts of E-Al_2_Y. Therefore, the as-cast YA65 alloy can be regarded as the Mg alloy containing P-Al_2_Y particles.

**Figure 4:**
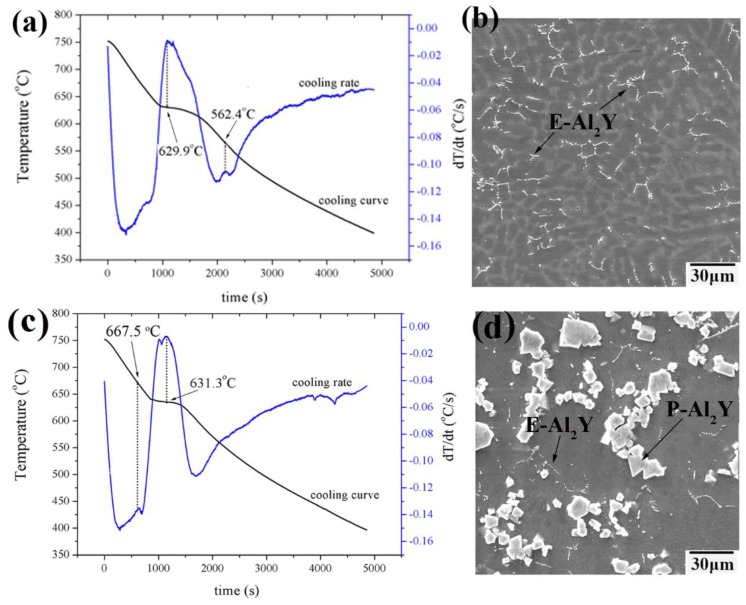
Thermal analysis results and solidification microstructures of Mg-6Y-*x*Al alloys: (**a**,**b**) YA605; (**c**,**d**) YA65.

In order to further certify the precipitation sequence of the two types of Al_2_Y phases as shown above, the thermal analysis tests were conducted for the as-cast YA605 and YA65 alloys, respectively. There are detailed descriptions and experimental verification of the test previously reported in the literature [26,27]. Figure 4 shows the cooling rate and cooling curves of alloy solidification process and the corresponding microstructure of the alloy at room temperature. The only one main peak can be clearly observed in the cooling rate curve in both alloys (Figure 4a,c). At the same time, an obvious plateau can be seen in the cooling curve, implying a major phase transformation that is essentially the formation of α-Mg. It indicates that the formation temperature of α-Mg is 629.9 °C for the YA605 alloy, while the temperature is 631.3 °C for the YA65 alloy. Chiu et al. reported that the endothermic peaks for the melting of Mg are at 632 °C in the Mg-Zn-Y alloy [27]. The microstructure of the thermal analysis (Figure 4b,d) and the as-cast (Figure 1a,f) two alloys is similar. After further analysis, we determined that the peak at 562.4 °C (Figure 4a) corresponds to the E-Al2Y phase precipitation in the YA605 alloy, while the peak at 667.5 °C (Figure 4c) corresponds to the P-Al_2_Y phase precipitation in the YA65 alloy. From the microstructural observations (Figure 4d), there is also a small amount of the E-Al_2_Y in the YA65 alloy but no exothermic peak was present in the cooling curve. This may be due to the fact that the relative content of E-Al_2_Y phase is very small in the YA65 alloy, as shown in Figure 1f and Figure 4d, since the main second phase of the YA65 alloy is P-Al_2_Y. Therefore, only the P-Al_2_Y phase precipitation peak was observed in the cooling rate curve for the YA65 alloy. The above analysis indicates that the P-Al_2_Y particles were formed prior to the solidification of α-Mg. This result is consistent with that of Chang et al. [22]. In addition, Liu et al. reported that the first precipitated phase in the solidification of the high-temperature liquid phase was the granulous Al_2_Y phase in the AZ61-Y alloy [28].

**Figure 5:**
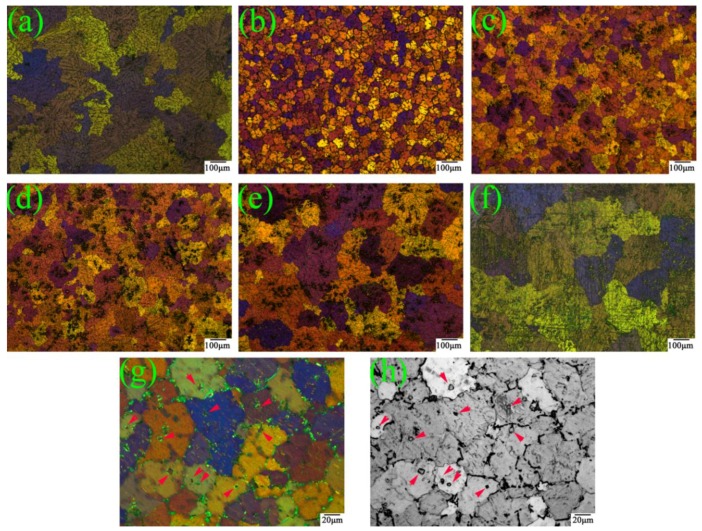
Optical micrographs of as-cast Mg-6Y alloys with the different addition Al contents: (**a**) 0.5%; (**b,g,h**) 1%; (**c**) 2%; (**d**) 3%; (**e**) 4%; (**f**) 5%.

**Figure 6:**
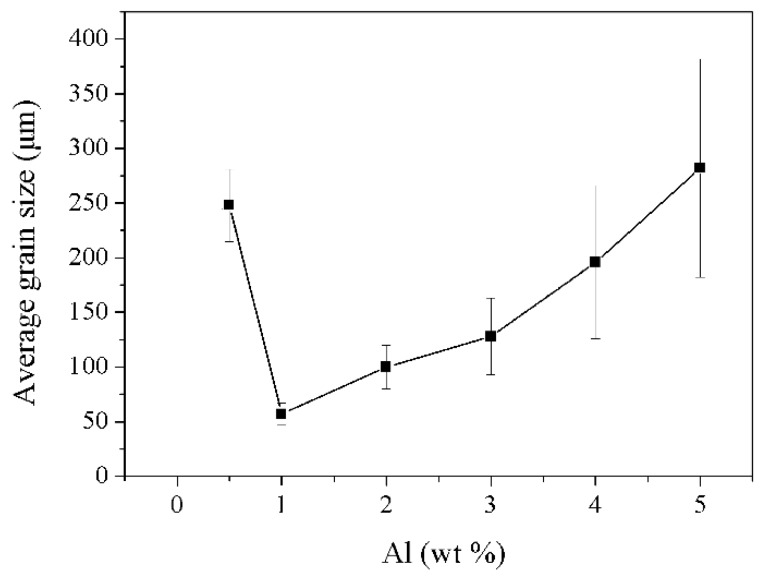
Grain size variation of the as-cast Mg-6Y alloys with additional amounts of the Al.

Figure 5 and Figure 6 show the optical micrographs of the as-cast Mg-6Y alloys with different Al content and the variation of the average grain sizes, respectively. It is obviously shown that the grain size first decreases before subsequently increases with increasing content of Al. When the proportion of the additional Al is 1%, the average grain size is greatly refined to be about 57 μm, which was the smallest grain size. The grain morphology of the YA61 alloy is equiaxed and it is easily observed that there are some particles (represented by arrows in Figure 5b,g,h) at the grain center in the optical microscope. A similar phenomenon has been found in the literature [20,21,23]. However, as the content of Al continued to increase by 2–5%, the grain gradually coarsened. In particular, the grain size of the as-cast YA65 alloy with a coarse dendritic structure is larger than that of the as-cast YA605 alloy.

According to the above analysis of the grain size and microstructure of the as-cast alloys, the main reason for grain refinement in Mg-6Y alloys by adding 1% Al is that the P-Al_2_Y particles with a uniform distribution and size of 4–6 μm were reproducibly observed at the centers of many refined grains of the YA61 alloy. This result is consistent with the grain refinement of the Mg-10Y-1Al [23]. The reason for the grain refinement is the heterogeneous nucleation of the primary α-Mg on P-Al_2_Y particles that are not on the E- Al_2_Y phase. It is indicated that adding Al into the as-cast Mg-6Y alloy can also promote the in-situ formation of P-Al_2_Y particles with heterogenous nucleation. However, when the number of the P-Al_2_Y particles increases, the grain size will not be refined but becomes more coarse, as shown in Figure 5. This phenomenon has not been reported in previous studies. In general, the grain refinement of polycrystalline materials is mainly determined by enhancing the nucleation rate in the melt and restraining grain growth. Thus, the main approaches for grain refinements involve adding potent nucleating agents or solute elements in order to lead to the constitutional undercooling. In our work, the nucleating agent is P-Al_2_Y and the solute elements are Y and Al. However, the number of potent P-Al_2_Y particles remarkably decreases due to its aggregation and size increase compared to that of YA61 alloy (as shown in Figure 1b,f). At the same time, the solid solution elements decrease due to the formation of stable Al_2_Y compounds. Easton et al. [29,30] also reported that the grain size is not only related to the heterogeneous nucleation sites but also the solute. The two factors mentioned above result in grain coarsening so the grain size of the as-cast YA65 alloy is bigger. Those results demonstrate that there are only P-Al_2_Y particles but less solute content, which means that we cannot refine gains by increasing the nucleation sites in the solidification process. However, it is possible for the YA65 alloy (the Mg alloy containing P-Al_2_Y particles) to be added into the other magnesium alloys to refine grains as a grain refiner. Research will be conducted in the future.

### 3.2. Microstructure and Mechanical Properties of the As-Extruded YA65 Alloy

**Figure 7:**
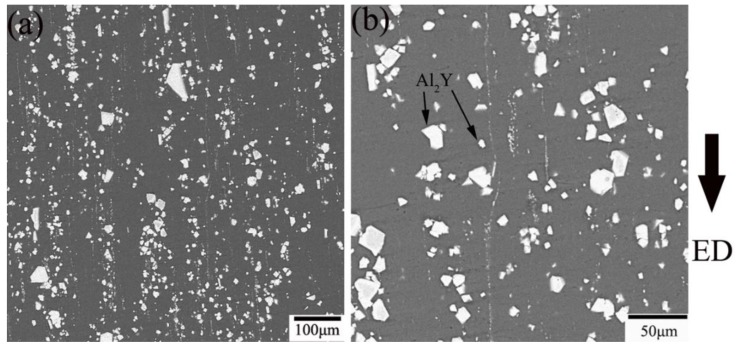
BSE-SEM images of the as-extruded YA65 alloy: (**a**) low magnification; (**b**) high magnification.

**Figure 8:**
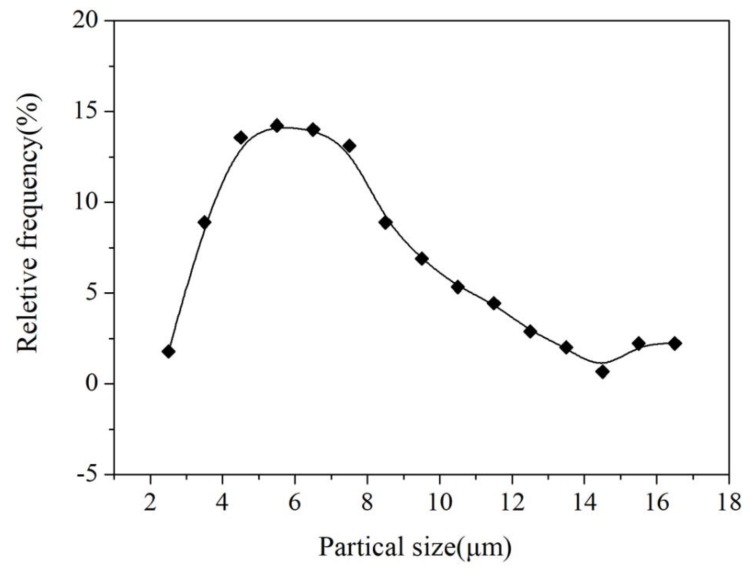
Size distribution of the Al_2_Y particles in the as-extruded YA65 alloy.

**Figure 9:**
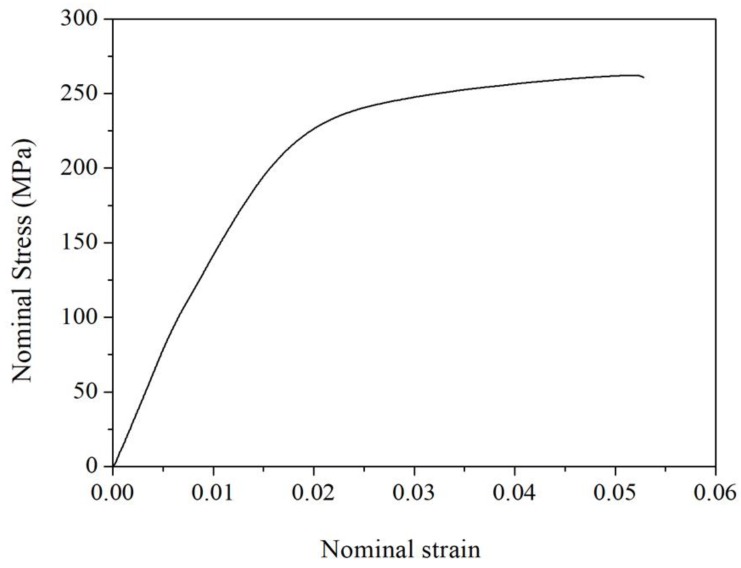
Tensile stress–strain curves of as-extruded YA65 alloy.

Figure 7 shows the microstructure of the YA65 alloy after hot extrusion, including the size and distribution of the P-Al_2_Y phase in the as-extruded alloy. It can be seen that the P-Al_2_Y phases existing in the as-extruded alloy are both broken and are uniformly distributed along the extrusion direction during hot processing compared with the state of the cast. However, the morphology of the P-Al_2_Y particles is still polygonal. In addition, the distribution of the particle sizes of the P-Al_2_Y phase in the as-extruded YA65 alloy is shown in Figure 8. There is a broad range for the size distribution of the P-Al_2_Y particles (3–17 μm). However, about 65 vol % of the particles are 4–7 μm, which is consistent with the requirement as a grain refiner, according to the active P-Al_2_Y nucleation particles in the Mg-10Y alloy [23], the active Al_2_Ce nucleation particles in the Mg-6Ce alloy [20], the active Al_2_Gd nucleation particles in the Mg-10Gd alloy [18] and the active Al_2_Nd nucleation particles in the Mg-5Nd alloy [21]. In addition, the as-extruded YA65 alloy can also be regarded as magnesium matrix composites that are reinforced by the P-Al_2_Y particles. The mass percentage content of the P-Al_2_Y particles is 10%. In order to investigate the effects of P-Al_2_Y particles on the strength of the Mg matrix, the mechanical properties of the alloy were tested at ambient temperatures. Figure 9 shows the nominal tensile stress–strain curves of the as-extruded YA65 alloys at ambient temperatures and the detailed mechanical data are summarized in Table 3. Apparently, the reinforcement of P-Al_2_Y particles can improve the tensile properties of the Mg matrix remarkably compared to that of pure Mg [31,32]. Because the P-Al_2_Y particles are uniformly distributed in the Mg matrix (Figure 7), this can strengthen the Mg matrix effectively by dispersion strengthening.

## 4. Conclusions

In the present paper, the magnesium alloy containing the P-Al_2_Y particles is successfully fabricated by changing the content of Al in the Mg-6Y alloy melt. Its microstructure and mechanical properties are characterized and the main conclusions can be described as follows:

(1) There are two types of Al_2_Y phases in the as-cast Mg-6Y-xAl (x = 0.5–5) alloys. One is the pre-precipitated phase with a polygonal particle morphology and the other is the irregular eutectic phase. In addition, with an increase in Al content, the polygonal particles phase increases, while the discontinuous network phase reduces gradually.

(2) The grain size of the as-cast Mg-6Y alloy was refined dramatically by adding 1% Al. After adding more Al, the grain size obviously increases. The reasons were respectively attributed to P-Al_2_Y particles, which can act as heterogeneous nucleation sites during the solidification process with continuous decreases in P-Al_2_Y particles.

(3) In comparison with the as-cast conditions, the hot extrusion process causes the Al_2_Y particles to be refined and dispersed distribution in the in-situ Mg-6Y-5Al alloy, which can be regarded as the P-Al_2_Y particle-reinforced magnesium matrix composite. The reinforcements of Al_2_Y particles can improve the tensile properties of the Mg matrix remarkably, while the elongation decreases at ambient temperatures.

## Figures and Tables

**Table 1:** Chemical compositions of the Mg-6Y-*x*Al alloys (wt %).

Alloy No.	Y	Al	Mg
YA605	6.21	0.46	Balance
YA61	6.18	1.03	Balance
YA62	6.15	1.78	Balance
YA63	6.20	3.12	Balance
YA64	6.22	3.85	Balance
YA65	6.25	4.9	Balance

**Table 2:** The EDS results of solution yttrium from the Mg-6Y-*x*Al matrix in Figure 1.

Alloys	Y605	Y61	Y62	Y63	Y64	Y65
Solution Yttrium (wt %)	3.25	3.3	2.34	1.38	0.53	0.18

**Table 3:** Tensile properties of the as-extruded YA65 alloy at room temperature (UTS: ultimate tensile strength; TYS: yield tensile strength; FE: elongation).

Alloys	UTS (Mpa)	TYS (Mpa)	FE (%)	Reference
Extruded-YA65 (Mg-10Al_2_Y)	263 ± 2	200 ± 5	3.4 ± 0.5	This study
Extruded-Pure Mg	208	120	12	[31]
Extruded-Pure Mg	183	112	13	[32]
